# The prevalence of obstructive sleep apnea in Japanese asthma patients

**DOI:** 10.1186/s13223-024-00875-x

**Published:** 2024-02-03

**Authors:** Mina Yasuda, Kazunori Tobino, Norihiro Harada, Ryunosuke Ooi, Takuto Sueyasu, Saori Nishizawa, Miyuki Munechika, Kohei Yoshimine, Yuki Ko, Yuki Yoshimatsu, Kosuke Tsuruno, Hiromi Ide, Kazuhisa Takahashi

**Affiliations:** 1 Department of Respiratory Medicine, Iizuka Hospital, 3-83 Yoshio Iizuka, Fukuoka, 820-8505 Japan; 2 Department of Respiratory Medicine, Juntendo University Faculty of Medicine and Graduate School of Medicine, Tokyo, Japan

**Keywords:** Obstructive sleep apnea, Bronchial asthma, Japanese

## Abstract

**Background:**

Obstructive sleep apnea (OSA) occurs more commonly in asthma patients than in the general population because these conditions share some comorbidities. In Japan, the prevalence of OSA in the general population is reported to be approximately 20%; however, few reports have described the prevalence of OSA in asthma patients. Furthermore, the characteristics of Japanese patients with OSA and asthma are not clear.

**Methods:**

Adult asthma patients were recruited from the outpatient departments of our institution between August 31, 2017, and March 31, 2019. In all included patients, the presence and severity of OSA were evaluated by the Epworth Sleepiness Scale (ESS) and a home sleep test (HST) using portable polysomnography (PSG). The rate of coexisting OSA in asthma patients and the characteristics of those patients according to the severity of OSA were investigated.

**Results:**

Fifty-three patients were included. OSA was detected in 36 (67.9%) patients (mild, *n* = 15; moderate, *n* = 14; and severe, *n* = 7). Patients with OSA had significantly higher body mass index, Brinkman index, apnea-hypopnea index (AHI), and 3% oxygen desaturation index (ODI) values in comparison to those without OSA, while the percentage of the predicted value of forced vital capacity (%FVC) and lowest SpO_2_ levels were significantly lower. As the severity of OSA increased, age, brain natriuretic peptide level, AHI, and 3%ODI increased, and in contrast, FVC, %FVC, forced expiratory volume in one second (FEV_1_), percentage of the predicted value of FEV_1_ (%FEV_1_), Epworth Sleepiness Scale (ESS), 3%ODI, and lowest SpO_2_ levels decreased. In particular, the fact that the ESS value was inversely correlated with the severity of OSA in our patients was different from the general characteristics of OSA. Moreover, the AHI value was negatively correlated with FVC, %FVC, FEV_1_, and %FEV_1_. BMI was the only independent factor for the presence of OSA, and for asthma severity (FEV1, % of predicted), there was a weak correlation with smoking history.

**Conclusions:**

This is the first report to investigate the prevalence of OSA in Japanese asthma patients, using an HST. This study suggests that an HST should be performed in addition to the sleep interview for asthma patients with refractory disease, a low pulmonary function, advanced age, and high BMI because the more severe the OSA, the lower the ESS value may be.

## Introduction

Asthma is a heterogeneous disease, usually characterized by chronic airway inflammation. It is defined by the history of respiratory symptoms, such as wheezing, shortness of breath, chest tightness, and cough—which vary over time and in intensity—together with variable expiratory airflow limitation [1]. Recently, the use of biological agents, such as omalizumab, mepolizumab, benralizumab, and dupilumab, has been approved for severe asthma in Japan, which has led to the improvement of asthma treatment [2–5]. However, there are some patients whose disease is poorly controlled, even after such treatment. Hekking et al. revealed that 3.6% of asthma patients over 18 years of age met the definition of severe asthma [6]. In such cases, it is necessary to search for comorbidities that make it difficult to control asthma, such as anxiety, depression, obesity, deconditioning, chronic rhinosinusitis, vocal cord dysfunction, gastro-esophageal reflux (GER), chronic obstructive pulmonary disease (COPD), obstructive sleep apnea (OSA), bronchiectasis, cardiac disease, and kyphosis due to osteoporosis [7].

OSA is a sleep-related breathing disorder; its prevalence is currently increasing [8–10]. OSA results in periodic narrowing and obstruction of the pharyngeal airway during sleep. When untreated, it has been reported to be associated with cardiovascular disease, metabolic disorders, cognitive dysfunction, depression, lost productivity, and workplace and motor vehicle accidents [11–18]. The most common symptoms of OSA are daytime sleepiness, fatigue, nocturia, morning headache, and memory loss [19, 20]. OSA occurs more commonly in asthma patients in comparison to the general population because these conditions share some comorbidities, such as obesity, rhinitis, and GER [21]. In fact, the prevalence of OSA in the general population, non-severe asthma patients, and severe asthma patients are reported to be approximately 9–38% [22], 19–60% [23–25], and 95% [26, 27], respectively. These data suggest the importance of searching for OSA in asthma patients. In clinical practice, OSA is easily missed in asthma patients because polysomnography (PSG), which is time consuming and costly, is required for the diagnosis, and both doctors and patients tend to think that nighttime symptoms are caused by asthma. In Japan, the prevalence of OSA in the general population is reported to be approximately 20% [28, 29]; however, few reports have describe the prevalence of OSA in asthma patients. In this study, we investigated the prevalence of OSA in Japanese asthma patients using a home sleep test (HST) with portable PSG and evaluated the patient characteristics.

## Methods

### Patients

**Fig. 1:**
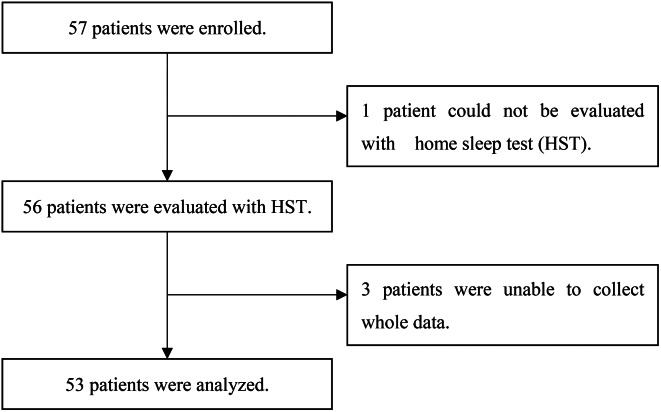
Consolidated Standards of Reporting Trial diagram showing study progression. Fifty-three patients were included in the present study

Consecutive patients with asthma, who were 20 years of age or older, who gave their informed consent, were recruited from our outpatient clinic at Iizuka Hospital (Fukuoka, Japan) between August 31, 2017, and March 31, 2019. The diagnosis of asthma and disease severity were assessed according to the Global Initiative for Asthma (GINA) guidelines [1]. Patients who met any of the following criteria were not enrolled in the study : coexistence of any respiratory disease other than asthma, uncontrolled heart failure, neuromuscular disease. Patients who could not be evaluated a home sleep test and had incomplete data were excluded from the study (Fig. 1).The present study was reviewed and approved by Iizuka Hospital Ethics Committee (AIH-17,120). This study was registered in the UMIN Clinical Trial Registry (UMIN000034263) (http://www.umin.ac.jp/).

### Measurements

All included patients were evaluated by the Epworth Sleepiness Scale (ESS) and an HST using a portable PSG (Alice NightOne^®^, Koninklijke Philips N.V.) for the presence and severity of OSA [30]. The diagnosis of OSA complied with the American Academy of Sleep Medicine, International classification of sleep disorders 3rd edition [31]. The apnea-hypopnea index (AHI) was defined as the number of apneas and hypopneas divided by total sleep time, and patients were classified as follows according to the AHI value: AHI 5 to < 15, mild; AHI 15 to < 30, moderate; and AHI ≥ 30, severe. We proposed continuous positive airway pressure (CPAP) treatment to patients with AHI ≥ 40 on a portable PSG or with AHI ≥ 20 on additional PSG, and only treated patients who gave their consent. The asthma control test (ACT) score, pulmonary function parameters, and fractional exhaled nitric oxide (FeNO) levels (NIOX VERO, Chest M.I., Tokyo, Japan) were also measured for the assessment of asthma in all included patients. The coexistence of rhinitis and GER were screened using the Self-assessment of Allergic Rhinitis and Asthma (SACRA) questionnaire [32] and the Frequency Scale for the Symptoms of GERD (FSSG), respectively [33]. Spirometry was performed with a Chestac-8900 spirometer (Chest Co., Ltd., Tokyo, Japan) and followed the standards of the Japanese Respiratory Society [34]. Other clinical and laboratory data, including plasma brain natriuretic peptide (BNP) levels, were extracted from the patients’ medical records.

### Statistical analysis

The differences in parameters between groups were examined using Fischer’s exact test and the Mann-Whitney U-test. Differences among more than two groups were analyzed for significance using the Kruskal–Wallis test. A post-hoc test was performed in Turkey’s pairwise comparison test. For correlation between variables, Pearson’s correlation coefficient and Spearman’s rank correlation coefficient were used, where appropriate. We performed a logistic regression analysis to examine confounding factors in the association between the presence of OSA and asthma severity. All reported P values are 2-sided, and P values of < 0.05 were considered statistically significant. Statistical analyses were performed using the EZR software program (Saitama Medical Center, Jichi Medical University), which is a graphical user interface for the R software program (The R Foundation for Statistical Computing, version 2.13.0) [35].

## Results

### Patient characteristics

Fifty-three patients (male, *n* = 13; female, *n* = 40) were included in the present study (Table 1). The mean age and body mass index (BMI) were 59 years and 26.4 kg/m^2^, respectively. The number of patients at each GINA treatment step was as follows: 0 in step 1; 1 (1.9%) in step 2; 16 (30.2%) in step 3; 26 (49.1%) in step 4; and 10 (18.9%) in step 5. The mean fractional exhaled FeNO, percentage of the predicted value of forced vital capacity (%FVC), and percentage of the predicted value of forced expiratory volume in one second (%FEV_1_) was 39.8 ppb, 99.7%, and 85.8%, respectively. Twenty-five (47.2%) patients had allergic rhinitis.

**Table 1:** Patient characteristics

**Characteristics**	
Male/Female, n (%)	13 (24.5)/40 (75.5)
Age, years	59.0 ± 16.4
Age at asthma onset, years	36.8 ± 22.9
BMI, kg/m2	26.4 ± 6.2
Smoking status: Never/Ex./Current, n (%)	29 (54.7)/14 (26.4)/10 (18.9)
Smoking history, pack-year	12.1 ± 18.9
Hypertension, n (%)	19 (35.8)
Diabetes, n (%)	14 (26.4)
BNP, pg/ml	40.4 ± 79.3
GINA step 1/2/3/4/5, n (%)	0/1/16/26/10 (0/1.9/30.2/49.1/18.9)
Allergic rhinitis, n (%)	25 (47.2)
FSSG score	8.13 ± 9.0
ACT score	21.6 ± 3.8
ACQ-6	0.9 ± 0.8
FeNO (ppb)	39.8 ± 38.6
Peripheral blood neutrophil count, cells/µL	4198.7 ± 1898.6
Peripheral blood eosinophil count, cells/µL	377.0 ± 762.6
Serum IgE level, IU/mL	820.6 ± 1997.5
FVC, L	2.8 ± 0.7
FVC, % of predicted	99.7 ± 15.0
FEV_1_, L	2.0 ± 0.6
FEV_1_, % of predicted	85.9 ± 18.4
FEV_1_/FVC, %	70.8 ± 12.1
MMF, L	1.4 ± 1.1
MMF, % of predicted	47.2 ± 30.4

### Results of ESS and PSG

The mean values of ESS, AHI, and 3% oxygen desaturation index (ODI) were 7.3 (range, 0–22), 16.3 (range, 0.3–69.3), and 16.0 (range, 1.1–85.1), respectively (Table 2). OSA was detected in 36 (67.9%) patients (mild, *n* = 15; moderate, *n* = 14; and severe, *n* = 7).

**Table 2:** Results of Epworth Sleepiness Scale and polysomnography

Measurements	Value
Epworth Sleepiness Scale	7.3 ± 4.6
Apnea Hypopnea Index	18.7 ± 16.9
3% oxygen desaturation index	18.3 ± 18.5
Lowest SpO_2_	82.2 ± 9.0
Number of patients with OSA Mild Moderate Severe	36 15 14 7

Data are presented as the mean ± standard deviation

### Comparison between patients with and without OSA

Patients with OSA had significant higher BMI, Brinkman index, AHI, and 3% ODI values than those without OSA, while %FVC and lowest SpO_2_ levels were significantly lower (Table 3). No significant differences were found in the other patient characteristics, including GINA steps and ESS, of the two groups.There was a statistically significant difference in FVC, % of predicted and FEV1, % of predicted between the groups with and without OSA, suggesting an association between OSA and asthma severity (Table 3). Furthermore, BMI and Brinkman Index, which are generally considered to be related to these two items, also showed statistically significant differences between groups. Therefore, to examine confounding factors in the association between the presence or absence of OSA and asthma severity, we performed logistic regression analysis with OSA as the objective variable and age, gender, BMI, Brinkman Index, FVC, % of predicted, and FEV1, % of predicted as explanatory variables. As a result, BMI was the only statistically independent variable related to the presence or absence of OSA (odds ratio, 1.21; 95% confidence interval, 1.04–1.42; *p* = 0.02) (Table 4). When correlations between explanatory variables were examined, a statistically significant weak negative correlation (spearman’s rank correlation, *r* = -0.357, *p* = 0.009) was found only between the Brinkman Index and FEV1, % of predicted. In summary, BMI was the only independent factor for the presence of OSA, and for asthma severity (FEV1, % of predicted), there was a weak correlation with smoking history.

**Table 3:** Comparison between patients with and without OSA

	Without OSA (*n* = 17)	With OSA (*n* = 36)	P value
Male/Female, n (%)	2/15	11/25	0.25
Age, years	59.8 ± 16.8	58.7 ± 16.5	0.83
Age at asthma onset, years	30.5 ± 22.8	58.7 ± 16.5	0.16
BMI, kg/m^2^	23.1 ± 3.85	27.9 ± 6.6	< 0.01
Smoking status: Never/Ex./Current, n (%)	13 (76.5)/2 (11.8)/2 (11.8)	16 (44.5)/12 (33.3)/8 (22.2)	0.29
Brinkman Index	147.1 ± 398.6	290.5 ± 365.3	0.03
Hypertension, n (%)	3 (17.6)	16 (44.4)	0.07
Diabetes, n (%)	2 (11.8)	12 (33.3)	0.18
BNP, pg/ml	28.6 ± 48.3	43.0 ± 82.9	0.17
GINA step (1/2/3/4/5), n (%)	0/0/7 /7 /3	0/1/9/19/7	0.84
Allergic rhinitis, n (%)	8 (47.1)	17 (47.2)	0.78
FSSG score	6.82 ± 6.48	8.75 ± 9.94	0.76
ACT score	22.4 ± 3.2	21.6 ± 3.7	0.31
ACQ-6	0.69 ± 0.83	1.09 ± 0.8	0.06
FeNO (ppb)	37.7 ± 28.3	40.9 ± 43.2	0.71
Peripheral neutrophil count, cells/µL	3603.5 ± 1468.4	4479.7 ± 2028.9	0.14
Peripheral eosinophil count, cells/µL	582.1 ± 1311.6	280.2 ± 218.0	0.57
Serum IgE level, IU/mL	583.6 ± 1359.5	603.84 ± 744.74	0.32
FVC, L	2.77 ± 0.62	2.84 ± 0.67	0.65
FVC, % of predicted	106.1 ± 15.7	96.7 ± 13.9	0.02
FEV_1_, L	2.0 ± 0.7	1.99 ± 0.59	0.8
FEV_1_, % of predicted	92.8 ± 19.1	82.4 ± 17.8	0.05
FEV_1_/FVC, %	72.1 ± 12.8	70.1 ± 11.9	0.58
MMF, L	1.5 ± 1.4	1.36 ± 0.87	0.92
MMF, % of predicted	51.8 ± 39.6	45.1 ± 25.4	0.46
Epworth Sleepiness Scale	6.35 ± 4.18	7.78 ± 4.83	0.27
Apnea Hypopnea Index	5.0 ± 3.2	21.7 ± 16.0	< 0.01
3% oxygen desaturation index	5.2 ± 3.1	22.2 ± 18.4	< 0.01
Lowest SpO_2_	86.9 ± 8.7	80.0 ± 8.5	< 0.01

Data are presented as the mean ± standard deviation unless otherwise indicated BMI: body mass index, BNP: brain natriuretic peptide, GINA: global initiative for asthma, FSSG: frequency scale for the symptoms of gerd, ACT: asthma control test, ACQ: asthma control questionnaire, FeNO: fractional exhaled nitric oxide, IgE: imuunoglobulin E, FVC: forced vital capacity, FEV_1_: forced expiratory volume in one second, MMF: mid-maximal flow rate

**Table 4:** Results of logistic regression analysis on the presence or absence of OSA

	OR	95% CI	P value
Age (years old)	0.989	0.945–1.04	0.64
Sex	5.290	0.632–44.2	0.12
BMI (kg/m^2^)	1.210	1.040–1.42	0.02
Brinkman Index	1.000	0.997-1.00	0.81
FVC, % of predicted (%)	0.980	0.914–1.05	0.56
FEV1, % of predicted (%)	0.986	0.929–1.05	0.64

BMI: body mass index, FVC: forced vital capacity, FEV1: forced expiratory volume in one second, OR odds ratio, CI: confidence interval

### Patient characteristics according to severity of OSA

**Fig. 2:**
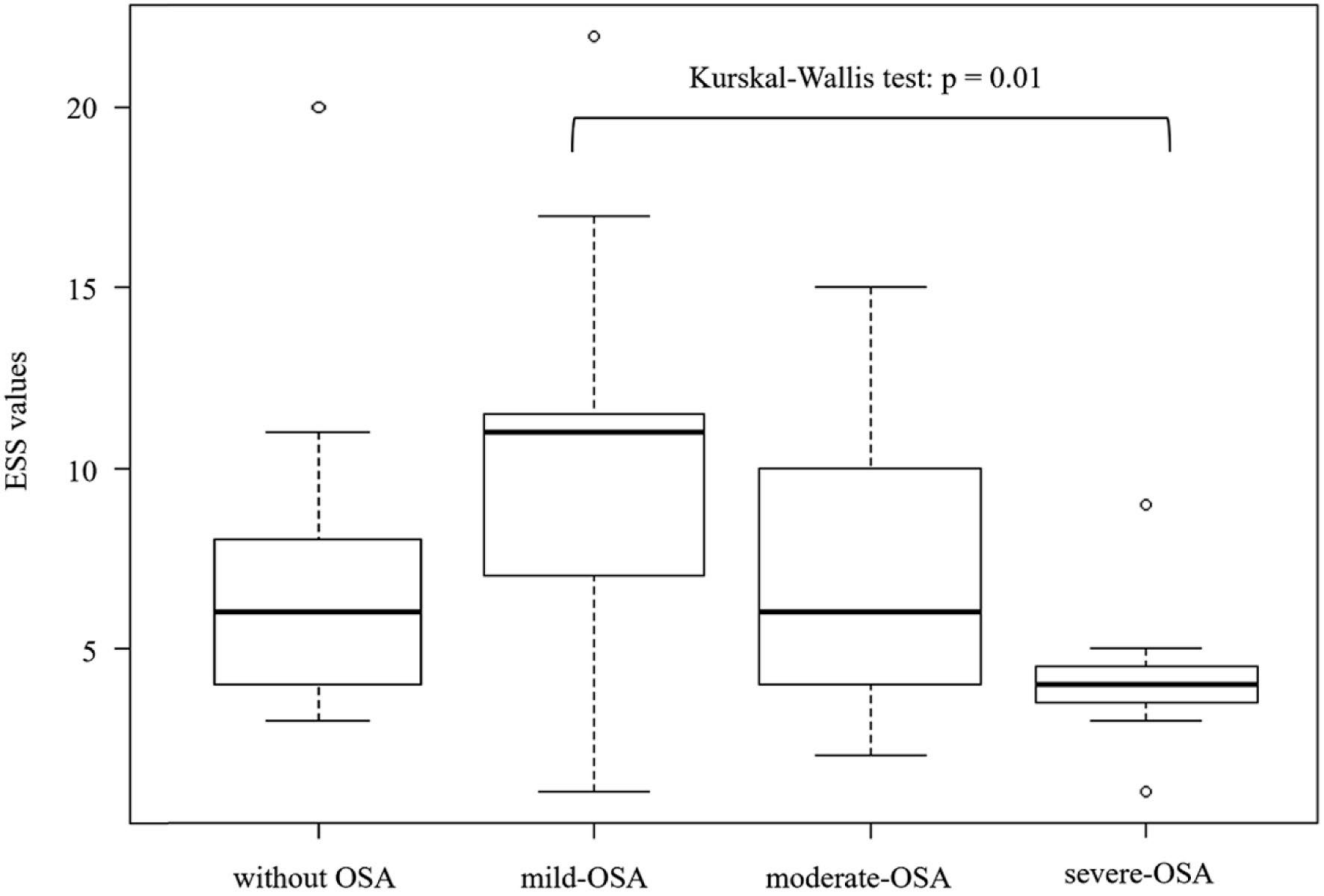
Epworth Sleepiness Scale (ESS) values according to the severity of obstructive sleep apnea (OSA). The ESS value was inversely correlated with the severity of OSA

**Fig. 3:**
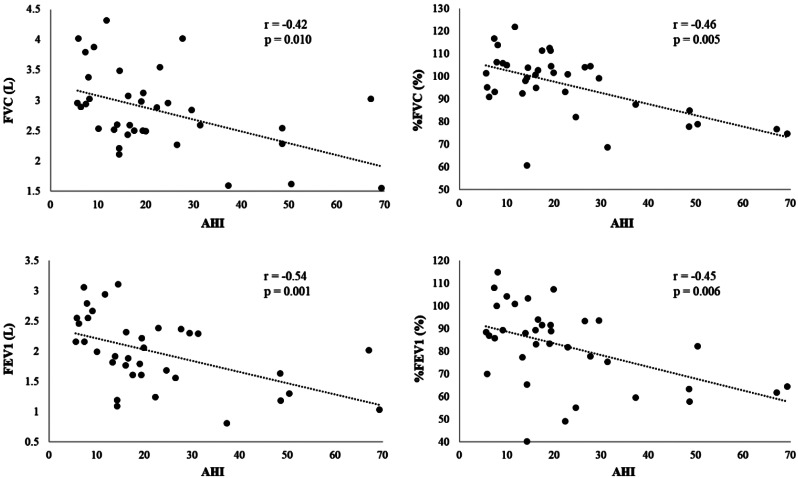
Relationships between AHI and pulmonary function test parameters (FVC, %FVC, FEV_1_, and %FEV_1_). The AHI value was negatively correlated with FVC, %FVC, FEV_1_, and %FEV_1_

When the patients were grouped according to the severity of OSA, significant differences were observed in age, BNP, FVC, %FVC, FEV_1_, %FEV_1_, ESS, AHI, 3%ODI, and lowest SpO_2_ level (Table 5). As the severity of OSA increased, age, BNP, AHI, and 3%ODI increased, while FVC, %FVC, FEV_1_, %FEV_1_, ESS, and lowest SpO_2_ decreased. The fact that the ESS value was inversely correlated with the severity of OSA in our patients was different from the general characteristics of OSA (Fig. 2). Moreover, the AHI value was negatively correlated with FVC, %FVC, FEV_1_, and %FEV_1_(Fig. 3).

**Table 5:** Comparison of patient characteristics by severity of OSA

	OSA severity			
Characteristics of asthma patients	Mild (*n* = 15)	Moderate (*n* = 14)	Severe (*n* = 7)	P value
Male/Female, n (%)	4/11	4/10	3/4	0.98
Age, years	49.9 ± 16.1	60.1 ± 13.1	74.7 ± 10.4	< 0.01
Age at asthma onset, years	29.5 ± 22.45	44.2 ± 16.2	54.3 ± 25.6	0.06
BMI, kg/m^2^	27.9 ± 8.16	26.5 ± 4.9	31.0 ± 5.30	0.34
Smoking status: Never/Ex./Current, n (%)	8/5/2	7/3/4	2/3/2	0.95
Brinkman Index	178.9 ± 213.8	361.9 ± 455.9	381.1 ± 411.5	1
Hypertension, n (%)	5 (33.3)	5 (35.7)	6 (85.7)	0.14
Diabetes, n (%)	2 (13.3)	4 (28.6)	5 (71.4)	0.08
BNP, pg/ml	17.1 ± 15.1	54.1 ± 120.2	87.5 ± 98.0	< 0.01
GINA step (1/2/3/4/5), n (%)	0/1/4/6/4	0/0/3/8/3	0/0/2/5/0	0.95
Allergic rhinitis, n (%)	10 (66.7)	4 (28.6)	3 (42.9)	0.24
FSSG score	11.93 ± 12.12	7.21 ± 8.16	5.00 ± 6.43	0.24
ACT score	20.8 ± 4.5	21.6 ± 3.7	21.4 ± 3.6	0.98
ACQ-6	1.24 ± 1.1	2.33 ± 1.1	0.98 ± 0.72	0.96
FeNO (ppb)	42.4 ± 46.9	42.1 ± 46.2	34.8 ± 32.2	1
Peripheral neutrophil count, cells/µL	4738.0 ± 2389.4	4470.7 ± 1920.4	3944.3 ± 1483.0	0.8
Peripheral eosinophil count, cells/µL	257.1 ± 144.0	280.6 ± 208.9	328.8 ± 360.7	0.99
Serum IgE level, IU/mL	634.5 ± 755.2	384.35 ± 587.6	511.0 ± 623.2	0.09
FVC, L	3.11 ± 0.68	2.87 ± 0.48	2.17 ± 0.59	0.03
FVC, % of predicted	100.4 ± 14.2	101.8 ± 8.04	78.6 ± 6.38	< 0.01
FEV_1_, L	2.30 ± 0.62	1.91 ± 0.36	1.47 ± 0.54	0.01
FEV_1_, % of predicted	88.2 ± 19.2	84.3 ± 15.4	66.4 ± 9.00	0.01
FEV_1_/FVC, %	74.0 ± 12.1	67.3 ± 10.3	67.6 ± 13.8	0.22
MMF, L	1.71 ± 0.97	1.17 ± 0.56	0.98 ± 0.98	0.06
MMF, % of predicted	51.7 ± 26.0	41.9 ± 21.3	37.2 ± 31.6	0.22
Epworth Sleepiness Scale	10.33 ± 5.29	6.79 ± 3.85	4.29 ± 2.43	0.01
Apnea Hypopnea Index	10.0 ± 3.38	21.3 ± 4.42	47.1 ± 16.4	< 0.01
3% oxygen desaturation index	9.2 ± 3.5	20.4 ± 4.7	53.6 ± 17.6	< 0.01
Lowest SpO_2_	85.9 ± 2.9	80.1 ± 4.8	67.5 ± 8.1	< 0.01

Data are presented as the mean ± standard deviation unless otherwise indicated. 　 BMI: body mass index, BNP: brain natriuretic peptide, GINA: global initiative for asthma, FSSG: frequency scale for the symptoms of gerd, ACT: asthma control test, ACQ: asthma control questionnaire, FeNO: fractional exhaled nitric oxide, IgE: imuunoglobulin E, FVC: forced vital capacity, FEV_1_: forced expiratory volume in one second, MMF: mid-maximal flow rate, CPAP: Continuous positive airway pressure

## Discussion

To the best of our knowledge, this is the first study to demonstrate the prevalence of OSA in Japanese patients with asthma by HST. The results of this study showed that the frequency of OSA in Japanese asthma patients was 67.9%. Among our patients with OSA, the severity of OSA was mild in 41.7%, moderate in 38.9%, and severe in 19.4%. The frequency of OSA according each GINA step was as follows: step 3, 56.3%; step 4, 73.1%; and step 5, 70%. Previous studies have reported a high frequency of OSA in asthma patients, ranging from 19 to 60% in non-severe asthma and reaching 95% in severe asthma [21, 26, 27]. Our results were similar to results from other countries.

Asthma and OSA affect each other; several mechanisms have been reported to be involved [21, 36]. In asthma patients, increased airway resistance and negative pressure during inspiration caused by nasal obstruction due to coexistent allergic rhinitis and nasal polyps lead to an increase in upper airway collapse [37–39]. Another mechanism involves a decrease in the pharyngeal cross-sectional area due to inflammatory cell infiltration of the upper airway, fat deposits and muscle weakness on the pharyngeal wall because of steroid use, and obesity. On the other hand, the induction of inflammation by OSA may also affect the exacerbation of asthma. Repeated hypoxia due to OSA increases the C-reactive protein, interleukin-6, 8-isoprostane and tumor necrosis factor levels [40, 41]. In fact, several studies have reported the presence of bronchial inflammation with high percentages of neutrophils in induced sputum with OSA patients [42, 43]. It has been reported that airway inflammation caused by these mechanisms can cause the exacerbation of asthma [44]. Additionally, elevated leptin levels in OSA lead to increased bronchial airway hyperresponsiveness and inflammation, which might cause the exacerbation of asthma [45–47]. Furthermore, OSA is thought to cause the exacerbation of asthma through the following mechanisms: neuromechanical reflex bronchoconstriction, GER, the indirect effect on dyspnea of OSA-induced cardiac dysfunction, and weight gain [36, 48–50]. Brinke et al. demonstrated that OSA was significantly associated with the frequent exacerbation of asthma [51]. Actually, in the GINA guidelines, OSA is described as a comorbidity that should be confirmed in patients with difficult-to-treat asthma [1].

In our study, we found a statistically significant difference in OSA-related parameters, such as BMI and AHI, between asthmatic patients with and without OSA. The severity of asthma and airway inflammation (FeNO and peripheral blood eosinophil count) did not differ to a statistically significant extent between the two groups. In asthma patients with OSA, the severity of OSA was negatively correlated with pulmonary function test parameters (FVC, %FVC, FEV_1_, and %FEV_1_). Wang et al. reported that asthma patients with OSA showed a greater decline in FEV_1_ in comparison to those without OSA in an AHI-dependent manner, and Emilsson et al. reported an association between OSA symptoms and the pulmonary function decline in asthma patients [52, 53]. Our study showed similar results to their previous reports. Our study also found that asthma patients with moderate to severe OSA were older than those with mild OSA. With a cutoff value of AHI ≥ 15 events/h, the prevalence of OSA has been reported to be 6–17% in the general population and up to 49% in the elderly [54]. Therefore, the frequency of severe OSA is also expected to be higher in elderly patients with asthma. Moreover, while there have been diverse reports on the relationship between BNP levels and severity of OSA [55, 56],our study showed a positive correlation between the two. It has been reported that BNP levels tend to increase with age [57]. In our patients, those with more severe OSA were older, suggesting that this factor also contributes to elevated BNP levels.

CPAP treatment for OSA has been reported to provide benefits for asthma patients with OSA [58]. Furthermore, in a prospective study, Serrano-Pariente et al. demonstrated that asthma control, quality of life, and the pulmonary function improved at 6 months after starting CPAP (used CPAP > 4 h/day) in asthma patients with moderate to severe OSA [59]. In our study, 13 of 36 patients with OSA were eligible for CPAP, but only 7 (moderate, *n* = 3; severe, *n* = 4) patients received CPAP treatment. In addition, 2 of the 7 patients discontinued CPAP treatment. The remaining 5 patients continued CPAP treatment and they did not experience any subsequent exacerbations of asthma during the course of the study (mean follow-up period, 6 months).

Questionnaires such as the ESS, pulse oximetry, and an HST with portable PSG have been recommended as screening methods for OSA. The most convenient of these tests is the questionnaire. Asthma patients with OSA were reported to have higher ESS scores than those without OSA [60, 61]. However, in our study, there was no statistically significant difference in the ESS values of patients with and without OSA, which is different from previous reports. Furthermore, the ESS values were decreased as OSA became more severe. A possible explanation for these results is that the patients with severe OSA in our study were older than the other patients, which may have affected their perception of their symptoms. In addition, since the duration of asthma was longer in patients with severe OSA, it is possible that these patients were less likely to notice the symptoms of OSA because they were accustomed to the symptoms of asthma. Based on the results of this study, we suggest that PSG should be considered in addition to ESS in asthma patients with refractory disease, a low pulmonary function, advanced age, and high BMI.

The present study was associated with several limitations. First, this was a single-center study and had no matched non-asthmatic controls. Second, the sample size was relatively small and the number of cases—when the patients were classified according to the severity of asthma—was insufficient. Third, in our study, the diagnosis of OSA was based on the results of HST for one night only [62]. Fourth, the Brinkman index values of OSA patients were higher than those of non-OSA patients, and more patients with COPD may have been included among the OSA patients. Both problems can only be solved by conducting a new multicenter study with a large study population. However, we believe that this study has demonstrated that screening for OSA using an HST is effective for picking up cases that cannot be detected by questionnaires alone.

## Conclusions

This is the first report to investigate the prevalence of OSA in Japanese patients with asthma, using an HST. This study suggests that HST should be performed in addition to the sleep interview for asthma patients with refractory disease, a low pulmonary function, advanced age, and high BMI because ESS values may decrease as OSA becomes more severe. Future trials to validate these results on a larger scale and to examine the effects of CPAP intervention are warranted.

## Data Availability

Not applicable.

## Abbreviations

OSA: obstructive sleep apnea
ESS: Epworth Sleepiness Scale
HST: home sleep test
AHI: apnea-hypopnea index
GER: gastro-esophageal reflux
COPD: chronic obstructive pulmonary disease
PSG: polysomnography
GINA: Global Initiative for Asthma
CPAP: continuous positive airway pressure
ACT: asthma control test
FeNO: fractional exhaled nitric oxide
SACRA: Self-assessment of Allergic Rhinitis and Asthma
FSSG: Frequency Scale for the Symptoms of GERD
BNP: brain natriuretic peptide
BMI: body mass index
FVC: forced vital capacity
FEV1: forced expiratory volume in 1 second
ODI: oxygen desaturation index
